# Quantification of host proteomic responses to genotype 4 hepatitis E virus replication facilitated by pregnancy serum

**DOI:** 10.1186/s12985-023-02080-5

**Published:** 2023-06-01

**Authors:** Zhongyao Qian, Chao Cong, Yi Li, Yanhong Bi, Qiuxia He, Tengyuan Li, Yueping Xia, Liangheng Xu, Houfack K. Mickael, Wenhai Yu, Jiankun Liu, Daqiao Wei, Fen Huang

**Affiliations:** 1 Medical School, Kunming University of Science and Technology, Kunming, People’s Republic of China; 2 Institute of Medical Biology, Chinese Academy of Medical Sciences and Peking Union Medical College, Kunming, People’s Republic of China; 3 920th Hospital of Joint Logistics Support Force of PLA, Kunming, People’s Republic of China

**Keywords:** Hepatitis E virus, Pregnancy, Proteomic analysis, iTRAQ, Virus-host interactions

## Abstract

**Background:**

Hepatitis E virus (HEV) infection is a common cause of acute hepatitis worldwide and causes approximately 30% case fatality rate among pregnant women. Pregnancy serum (PS), which contains a high concentration of estradiol, facilitates HEV replication in vitro through the suppression of the PI3K–AKT–mTOR and cAMPK–PKA–CREB signaling pathways. However, the proteomics of the complex host responses to HEV infection, especially how PS facilitates viral replication, remains unclear.

**Methods:**

In this study, the differences in the proteomics of HEV-infected HepG2 cells supplemented with fetal bovine serum (FBS) from those of HEV-infected HepG2 cells supplemented with serum from women in their third trimester of pregnancy were quantified by using isobaric tags for relative and absolute quantification technology.

**Results:**

A total of 1511 proteins were identified, among which 548 were defined as differentially expressed proteins (DEPs). HEV-infected cells supplemented with PS exhibited the most significant changes at the protein level. A total of 328 DEPs, including 66 up-regulated and 262 down-regulated proteins, were identified in HEV-infected cells supplemented with FBS, whereas 264 DEPs, including 201 up-regulated and 63 down-regulated proteins, were found in HEV-infected cells supplemented with PS. Subsequently, Gene Ontology and Kyoto Encyclopedia of Genes and Genomes analyses revealed that in HEV-infected cells, PS supplementation adjusted more host genes and signaling pathways than FBS supplementation. The DEPs involved in virus–host interaction participated in complex interactions, especially a large number of immune-related protein emerged in HEV-infected cells supplemented with PS. Three significant or interesting proteins, including filamin-A, thioredoxin, and cytochrome c, in HEV-infected cells were functionally verified.

**Conclusions:**

The results of this study provide new and comprehensive insight for exploring virus–host interactions and will benefit future studies on the pathogenesis of HEV in pregnant women.

**Supplementary Information:**

The online version contains supplementary material available at 10.1186/s12985-023-02080-5.

## Background

Hepatitis E virus (HEV), a single-stranded positive-sense RNA virus, is a predominant pathogen that is responsible for cases of acute hepatitis worldwide [1, 2]. HEV generally causes self-limiting acute hepatitis in healthy adults but pose a high risk of chronicity in immunocompromised individuals, such as organ transplant recipients [3] or HIV-infected patients [4]. Notably, pregnant women are more sensitive to HEV infection than nonpregnant women (8.4% vs. 2.6%) [5]. The maternal case fatality rate among pregnant women with jaundice and acute HEV infection is higher than that among pregnant women infected with other hepatitis viruses, including hepatitis A virus (HAV), hepatitis B virus (HBV), and hepatitis C virus (HCV) (41% vs. 7%) [6]. In India, the maternal case fatality rate among HEV-infected pregnant women is 26.9% [7]. Although most cases of maternal deaths are reported in areas wherein HEV genotypes 1 and 2 are prevalent, adverse pregnancy outcomes, such as spontaneous abortion, premature delivery, and stillbirth, have been found in countries where HEV genotypes 3 and 4 are endemic [8]. Preterm delivery, premature rupture of membranes, neonatal jaundice and potential risk of developing hyperlipidemia were reported in pregnant women infected with genotype 4 HEV in China [8, 9].

Nevertheless, the pathogenesis of HEV in pregnant women remains unclear. During pregnancy, the increase in hormones, mainly estradiol and progesterone, may increase the susceptibility of pregnant women to viral infections. In vitro, HEV replication in cells supplemented with pregnancy serum (PS) is significantly enhanced compared with those supplemented with fetal bovine serum (FBS) or non-pregnant serum (NPS) [10]*.* Notably, the estradiol analog 17β-estradiol accelerates HEV replication in vitro via the suppression of the PI3K–AKT–mTOR and cAMPK–PKA–CREB signaling pathways [11, 12]. Furthermore, the level of progesterone is associated with the enhancement of viral replication through the interaction of HEV with progesterone receptor membrane component 1/2 [13].

Estradiol and progesterone are crucial for pregnancy maintenance, and maternal immune tolerance is essential for fetal rejection. The suppression of host immunity during pregnancy contributes to pathogen infections. Maternal immune tolerance with the rare expression of interferon-stimulated genes (ISGs) accounts for the longer viral duration and higher viral titers in HEV-infected pregnant rhesus macaques than in nonpregnant rhesus macaques [14]. HEV infection involves numerous host pathways, such as the PI3K–AKT–mTOR [12], cAMPK–PKA–CREB [11], and NF–κB [15] signaling pathways. Thousands of network interactions occur between a virus and its host once infection occurs. Thus, comprehensive proteomic analysis must be performed to explore virus–host interactions.

Quantitative proteomics has been widely utilized to study differential protein expression patterns to reveal interactions between hosts and viruses, such as corona virus disease 2019 [16], HBV [17], and herpes simplex virus type 1 [18]. However, reports on the proteomic analysis of HEV infection, especially reports related to pregnancy, are rare. Isobaric tags for relative and absolute quantification (iTRAQ) is a powerful technology for quantifying proteomics and is especially useful for quantifying low-abundance proteins due to its higher sensitivity and precision than conventional proteomics methods [19]. In this work, HEV-infected cells supplemented with FBS or PS were collected for iTRAQ-based proteomics analysis to identify the virus–host protein regulation networks involved in HEV infection. Then, Western blot analysis was further performed for the functional validation of the three key proteins isolated through iTRAQ. This study provides new insight for exploring virus–host interactions and will promote further studies on the pathogenesis of HEV in pregnant women.

## Methods

### Ethics approval and consent to participate

All serum samples were collected from patients in Kunming, China. This study was approved by the medical ethics committee of the Medical Faculty, Kunming University of Science and Technology. Patients with HAV, HBV, HCV, and HIV were excluded. Written informed consent was obtained from the patients for the publication of this report and any accompanying images.

### Virus, cells and cell transfection

HEV-positive swine fecal samples containing HEV genotype 4 (GenBank accession no. KJ155502) were obtained from Kunming, China [20]. The fecal samples were converted into 10% (w/v) suspensions in DEPC–H_2_O and centrifuged at 12 000 × *g* at 4 °C for 10 min, filtered through 0.22 µm microfilters before viral inoculation, and treated with penicillin and streptomycin for 1 h. The suspensions were then stored in liquid nitrogen until use. Viral titers of 1.0 × 10^6^ copies/mL were determined by using quantitative Real-Time PCR (qRT-PCR) [21]. A human hepatoma cell line (HepG2 cells) and a human lung carcinoma cell line (A549 cells) were obtained from the American Type Culture Collection and maintained in Dulbecco’s modified Eagle’s medium containing 10% (v/v) FBS, 100 U/mL penicillin, and 100 µg/mL streptomycin at 37 °C under 5% CO_2_.

HepG2 cells were planted in 12-well microplates, incubated overnight to achieve 70%–80% confluence, washed twice with PBS, and transfected using Lipofectamine 3000 (ThermoFisher Scientific Inc., USA) following the manufacturer's instructions. The shRNA targeting FLNA were constructed according to the previous study [22]. Cells were transfected with shRNA-FLNA-1 plasmid to knock down FLNA.

### Virus inoculation

Thirty serum samples from women in the third trimester of pregnancy that were negative for HEV RNA, HEV IgG and HEV IgM were mixed together, filtered with a 0.22 mm microfilter and heat-inactivated at 56 °C for 30 min, defined as pregnancy serum (PS). Mixed serum samples from thirty healthy non-pregnant women defined as non-pregnant serum (NPS). The cells were planted in six-well microplates for 24 h before virus inoculation and supplemented with 10% FBS (HEV group), 10% NPS (HEV + NPS group), or 10% PS (HEV + PS group) at ~ 50% confluence. The protocol for HEV inoculation was performed in accordance with a previous study [23]. In brief, monolayer cells were washed thrice and inoculated with 0.2 mL of the filtered viral inoculum and 30 mM MgCl_2_ (final concentration) for 1 h. The solution was removed after inoculation, and fresh maintenance medium containing 2% FBS, 2% NPS, or 2% PS was added separately. The cells were collected either for proteomic analysis or viral replication determination at 6 days postinoculation (dpi).

### Protein preparation

Three biological replicates for each group were prepared for the iTRAQ-based proteomics experiments. The cells in Mock, HEV, and HEV + PS groups were suspended in the Lysis buffer (7 M Urea, 2 M Thiourea, 4% CHAPS, 40 mM Tris–HCl, pH 8.5, 1 mM PMSF, 2 mM EDTA) and sonicated in ice. The proteins were reduced with 10 mM DTT (final concentration) at 56 °C for 1 h and then alkylated by 55 mM IAM (final concentration) in the darkroom for 1 h. The reduced and alkylated protein mixtures were precipitated by adding 4 × volume of chilled acetone at − 20 °C overnight. After centrifugation at 4 °C, 30,000 *g*, the pellet was dissolved in 0.5 M TEAB (Applied Biosystems, Milan, Italy) and sonicated in ice. After centrifuging at 30 000 *g* at 4 °C, an aliquot of the supernatant was taken for determination of protein concentration. The proteins in the supernatant were kept at − 80 °C for further analysis.

### iTRAQ labeling and SCX fractionation

Total protein (100 μg) was taken out of each sample solution and then the protein was digested with Trypsin Gold (Promega, Madison, WI, USA) with the ratio of protein: trypsin = 30: 1 at 37 °C for 16 h. After trypsin digestion, peptides were dried by vacuum centrifugation. Peptides were reconstituted in 0.5 M TEAB and processed according to the manufacture’s protocol for 8-plex iTRAQ reagent (Applied Biosystems). Briefly, one unit of iTRAQ reagent was thawed and reconstituted in 24 μL isopropanol. Samples were labeled with the iTRAQ tags. The peptides were labeled with the isobaric tags, incubated at room temperature for 2 h. The labeled peptide mixtures were then pooled and dried by vacuum centrifugation. SCX chromatography was performed with a LC**-**20AB HPLC Pump system (Shimadzu, Kyoto, Japan). The iTRAQ**-**labeled peptide mixtures were reconstituted with 4 mL buffer A (25 mM NaH2PO4 in 25% ACN, pH 2.7) and loaded onto a 4.6 × 250 mm Ultremex SCX column containing 5-μm particles (Phenomenex). The peptides were eluted at a flow rate of 1 mL/min with a gradient of buffer A for 10 min, 5–60% buffer B (25 mM NaH2PO4, 1 M KCl in 25% ACN, pH 2.7) for 27 min, 60–100% buffer B for 1 min. The system was then maintained at 100% buffer B for 1 min before equilibrating with buffer A for 10 min prior to the next injection. Elution was monitored by measuring the absorbance at 214 nm, and fractions were collected every 1 min. The eluted peptides were pooled into 20 fractions, desalted with a Strata X C18 column (Phenomenex) and vacuum-dried.

### LC–ESI–MS/MS analysis

Each fraction was resuspended in buffer A (5% ACN, 0.1%FA) and centrifuged at 20000 *g* for 10 min, the final concentration of peptide was about 0.5 μg/μL on average. 10 μL supernatant was loaded on a LC-20AD nanoHPLC (Shimadzu, Kyoto, Japan) by the autosampler onto a 2 cm C18 trap column. Then, the peptides were eluted onto a 10 cm analytical C18 column (inner diameter 75 μm) packed in-house. The samples were loaded at 8 μL/min for 4 min, then the 35 min gradient was run at 300 nL/min starting from 2 to 35% B (95%ACN, 0.1%FA), followed by 5 min linear gradient to 60%, then, followed by 2 min linear gradient to 80%, and maintenance at 80% B for 4 min, and finally return to 5% in 1 min. Data acquisition was performed with a TripleTOF 5600 System (AB SCIEX, Concord, ON) fitted with a Nanospray III source (AB SCIEX, Concord, ON) and a pulled quartz tip as the emitter (New Objectives, Woburn, MA). Data was acquired using an ion spray voltage of 2.5 kV, curtain gas of 30 psi, nebulizer gas of 15 psi, and an interface heater temperature of 150. The MS was operated with a RP of greater than or equal to 30,000 FWHM for TOF MS scans. For IDA, survey scans were acquired in 250 ms and as many as 30 product ion scans were collected if exceeding a threshold of 120 counts per second (counts/s) and with a 2 + to 5 + charge-state. Total cycle time was fixed to 3.3 s. Q2 transmission window was 100 Da for 100%. Four time bins were summed for each scan at a pulser frequency value of 11 kHz through monitoring of the 40 GHz multichannel TDC detector with four-anode channel detection. A sweeping collision energy setting of 35 ± 5 eV coupled with iTRAQ adjust rolling collision energy was applied to all precursor ions for collision-induced dissociation. Dynamic exclusion was set for 1/2 of peak width (15 s), and then the precursor was refreshed off the exclusion list.

### Data analysis

Raw data were processed with Proteome Discoverer 2.1 (PD, Thermo Fisher Scientific Inc.) and submitted to iProx database (https://www.iprox.cn/page/home.html, IPX0004999000). Proteins identification were performed by using Mascot search engine (Matrix Science, London, UK; version 2.3.02). Proteins identified with *p* < 0.05 and fold changes (FC) > 1.2 were considered as DEPs. DEPs were hierarchically clustered with log2 fold-change (FC) log2(FC) values. Regulations of filamin-A (FLNA), thioredoxin (TXN) and cytochrome (CYCS) in HEV-infected HepG2 cells at 6 dpi were shown in Table 1 with fold changes.

**Table 1:** Regulation of three DEPs in HEV-infected HepG2 cells at 6 dpi as determined by iTRAQ

Gene symbol	Description	HEV versus Mock (Fold)	HEV + PS versus Mock (Fold)	HEV + PS versus HEV (Fold)
FLNA	Isoform 2 of filamin-A	0.546*	1.420*	2.264*
TXN	Thioredoxin	0.225*	0.997	3.258*
CYCS	Cytochrome c	0.136*	0.469*	3.920*

*Proteins with *p* < 0.05 and FC > 1.2 were considered to be significantly

### Gene Ontology function, Kyoto Encyclopedia of Genes and Genomes pathway, and protein–protein interaction network analyses

Gene Ontology (GO) and Kyoto Encyclopedia of Genes and Genomes (KEGG) pathway enrichment analyses were used to analyze the function of proteins (biological process, molecular function, and cellular component) and the involved pathway [24, 25]. The protein–protein interaction (PPI) networks of DEPs were analyzed by using STRNG database to obtain the global profile of HepG2 cells in response to HEV replication facilitated by PS [26]. Then, Cytoscape software version 3.0 was used to visualize PPIs [27].

### Western blot analysis

The expression levels of five key proteins in HepG2 cells were detected through Western blot analysis. Cells were collected at indicated times and lysed in radio-immunoprecipitation assay buffer (50 mM Tris, pH 7.4, 150 mM NaCl, 5 mM ethylenediaminetetraacetic acid, pH 8.0, 30 mM NaF, 1 mM Na_3_VO_4_, 40 mM β-glycerophosphate, 0.1 mM phenylmethylsulfonyl fluoride, protease inhibitors, 10% glycerol, and 1% Nonidet-P40). Proteins were analyzed through 10% sodium dodecyl sulfate–polyacrylamide gel electrophoresis and then transferred onto a nitrocellulose membrane. Nonspecific binding sites were blocked with 5% skimmed milk, and the membrane was separately incubated with primary antibodies, HEV ORF2 (Millipore, 1:1000 dilution), FLNA, CYCS, or TXN (Bioworld, 1:1000 dilution) at 4 °C overnight. Horseradish peroxidase-conjugated IgG was used as the secondary antibody (Promega, 1:10,000 dilution). The GAPDH protein served as the loading control. Bands were exposed to X-ray films by using an Immobilon ECL kit (Millipore).

### Gene quantification

Total RNA was isolated from cells by Trizol (Invitrogn, America) in accordance with the manufacturer's instructions. cDNA was prepared by using AMV Reverse Transcriptase XL (Takara, Japan) in accordance with the provided directions. The copy number of HEV was quantified through SYBR green-based qRT-PCR with HEV-specific primers as described in our previous study [21]. The expression levels of RIG-1 were quantified by using specific primers described in previous studies [28]. GAPDH was applied as the housekeeping control. Relative gene expression was calculated through the 2^−(△Ct of gene − △Ct of GAPDH)^ method, where Ct is the threshold cycle. qRT-PCR was performed with a Bio-Rad CFX96TM Real-Time PCR System.

### Statistical analysis

All experiments were performed at least three times. Data were presented as mean ± SD. Statistical analysis was performed on Western blot analysis results by using GraphPad Prism software version 8.0, and *p* values were calculated by using Student’s *t*-test to determine the significance of differences between two or more groups. *p* < 0.05 was considered to indicate statistical significance (Additional file 1).

## Results

### Identification of the acceleration of HEV replication in cells supplemented with PS

**Fig. 1:**
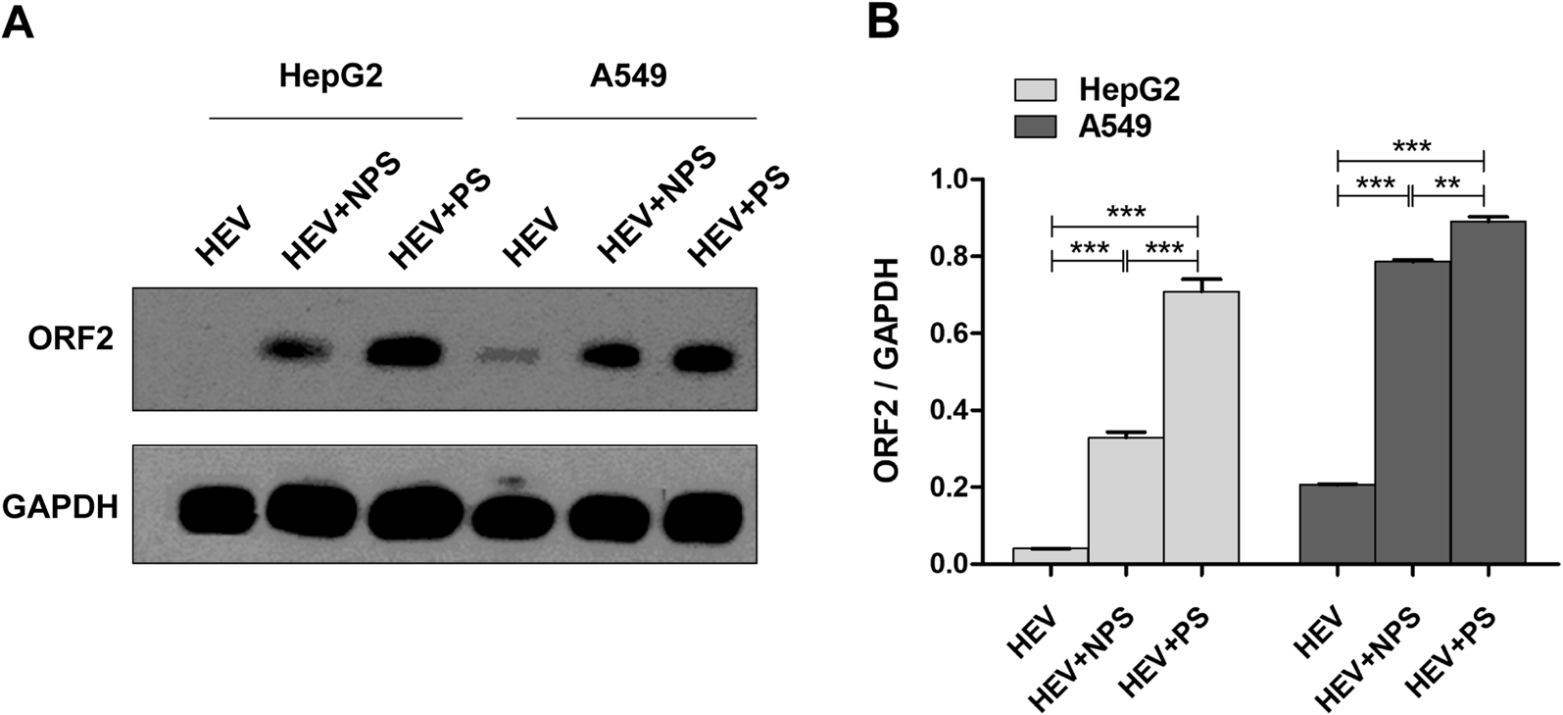
PS facilitates HEV replication. **A** HEV capsid proteins (ORF2) were detected in HepG2 and A549 cells through Western blot analysis at 6 dpi. **B** The relative expression of ORF2 was analyzed and normalized to that of GAPDH by using Image J software. Student’s t-test was performed to analyze the difference between two groups. *p < 0.05, **p < 0.01, ***p < 0.001

Hormones, estradiol, and progesterone play vital roles in maintaining pregnancy. The viral titer in pregnant women or pregnant rhesus macaques is significantly higher than that in nonpregnant ones [14]. In vitro, HEV replication is significantly facilitated by PS, especially PS from women in the third trimester of pregnancy [10]. However, the mechanism underlying this enhancement remains unclear. HEV-infected hepatoma cells (HepG2) and lung cancer cells (A549) were supplemented with 10% FBS, 10% NPS, or 10% PS from women in the third trimester of pregnancy to explore the enhancement in HEV infection under PS supplementation. The capsid protein of HEV was detected by Western blot analysis at 6 dpi. Notably, viral replication in cells supplemented with PS was significantly enhanced relative to that in cells supplemented with FBS and NPS (Fig. 1A and B). HepG2 cells derived from the liver were chosen as the candidate cells for HEV infection to explore HEV pathogenesis through proteomics analysis.

### Identification of the global changes of proteins in HEV-infected cells supplemented with PS

HepG2 cells were collected at 6 dpi and analyzed by iTRAQ to identify the DEPs in mock and HEV-infected cells supplemented with FBS or PS. iTRAQ is a powerful technology for protein quantification that exhibits higher sensitivity and precision than conventional proteomics methods especially when used to quantify proteins with low abundance [29, 30]. iTRAQ has been widely used to explore the interactions between viruses and their hosts [30–32]. In the present study, the DEPs in cells with or without HEV infection and supplemented with FBS or PS were identified by using iTRAQ. A total of 18 909 spectra and 5322 peptides were identified with 4847 unique peptides and 1511 proteins. Among the 1511 proteins, 14%, 12%, 12%, 11%, 10%, and 18% had weights of 10–20, 20–30, 30–40, 40–50, 50–60, and > 100 KDa, respectively. Most of the identified proteins comprised less than 10 peptides. Additionally, 42.03% of the identified proteins showed more than 10% sequence coverage. The possible functions of these proteins were predicted and classified in accordance with the COG database. The five top functional categories identified through general functional prediction were post-translational modification; protein turnover and chaperones; translation, ribosomal structure, and biogenesis; energy production and conversion; and carbohydrate transport and metabolism.

### Proteome profiling of the DEPs in HEV-infected cells supplemented with PS

**Fig. 2:**
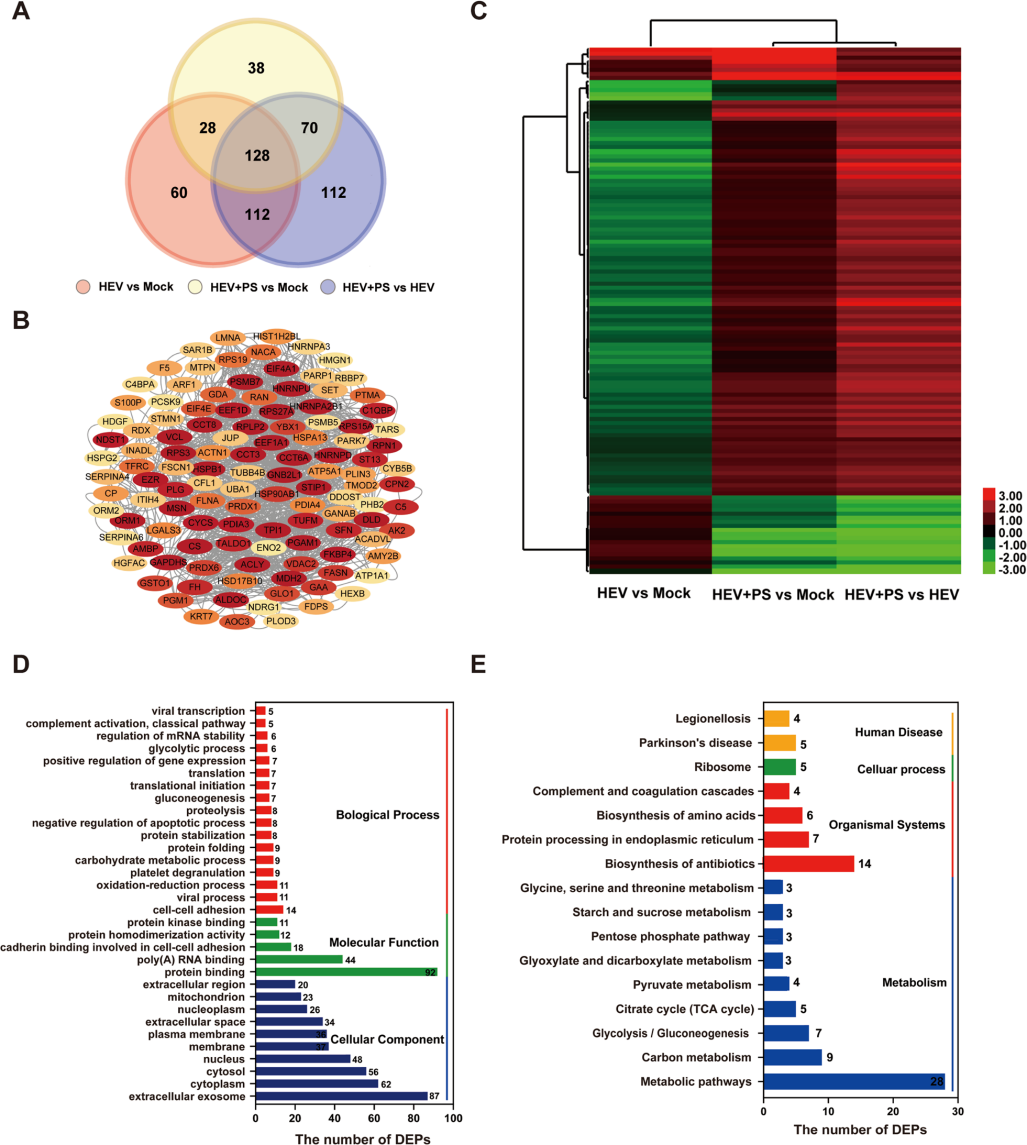
DEPs in HEV-infected cells supplemented with FBS or PS. **A** Venn diagram. The red circle indicates the DEPs in HEV-infected cells (HEV vs. Mock); the yellow circle indicates the DEPs in HEV-infected cells supplemented with PS (HEV + PS vs. Mock); and blue circles indicate the DEPs in HEV-infected cells supplemented with PS (HEV + PS vs. HEV). **B** PPI network of 110 dysregulated proteins among 128 DEPs. **C** Hierarchical clustering analysis of DEPs in cells with or without HEV infection and with PS or FBS supplementation. Clustering with log2(FC) values; red indicates up-regulated proteins, and green indicates down-regulated proteins. **D** Functional classification of the 128 dysregulated proteins by GO enrichment analysis. **E** KEGG pathway analysis was performed to investigate the significant pathways wherein the 128 proteins were enriched in accordance with the related networks for pathway mapping

In consideration of the alteration in host proteomic responses to HEV replication facilitated affected by PS, a Venn diagram displayed the differentially and coincidentally dysregulated proteins in HepG2 cells supplemented with PS (Fig. 2A). Among the total 1511 identified proteins, 548 were defined as DEPs. On the basis of the analysis, 60, 38, and 112 DEPs were exclusively identified with stage specificity in the HEV versus Mock, HEV + PS versus Mock, and HEV + PS versus HEV groups, respectively. In addition, a total of 128 proteins were found to be simultaneously regulated significantly. Interestingly, PPI analysis with STRING revealed that 118/128 DEPs formed a tight interaction network (Fig. 2B). Furthermore, clustering analysis was used to assess the relationships among 128 DEPs in HepG2 cells with or without PS supplementation. Notably, most of the DEPs were down-regulated in cells infected with HEV (Fig. 2C, HEV vs. Mock), whereas the inverse was observed in HEV-infected cells supplemented with PS (Fig. 2C, HEV + PS vs. mock). Significant changes occurred in HEV-infected cells supplemented with PS or FBS (Fig. 2C, HEV + PS vs. HEV). GO enrichment analysis was conducted to further predict the functions of 128 DEPs (Fig. 2D). Extracellular exosome was the most significantly enriched GO cellular component term, and protein binding was the most significantly enriched GO molecular function term. Meanwhile, biological process terms were mostly enriched in cell–cell adhesion. Notably, the identified DEPs were found to play important roles in viral processes, viral transcription, and complement activation. KEGG pathway analysis was also performed to investigate the significant pathways wherein the 128 DEPs were enriched (Fig. 2E). Most enriched KEGG pathways were mainly composed of metabolic pathways. The DEPs also played important roles in human diseases, such as legionellosis and Parkinson’s disease. Complement and coagulation cascades and antibiotic biosynthesis were also associated with the DEPs.

### Functional enrichment of the DEPs in HEV-infected cells supplemented with PS

**Fig. 3:**
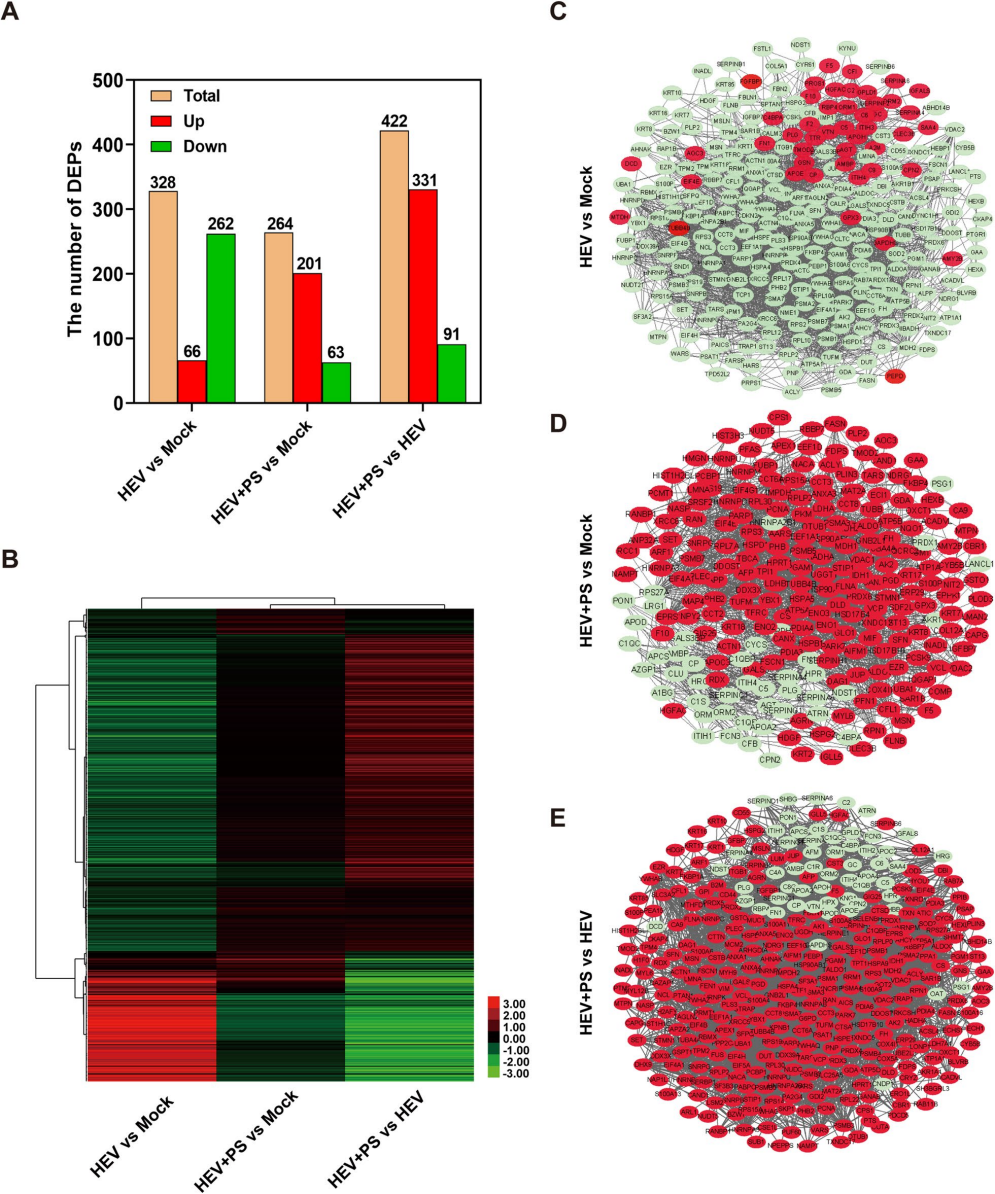
Comparison of DEPs in HEV-infected cells with or without PS supplementation. **A** Number of DEPs in different groups. **B** Hierarchical clustering analysis of 548 DEPs between different groups. Clustering with log2(FC) values; red indicates up-regulated proteins, and green indicates down-regulated proteins. PPI network of the DEPs in the HEV versus Mock (**C**), HEV + PS versus Mock (**D**), and HEV + PS versus HEV groups (**E**). PPI networks built by using the STRING database and Cytoscape software. Each node in the interaction network represents a DEP. Red nodes indicate up-regulated proteins, and green nodes indicate down-regulated proteins

A total of 328 proteins (66 up-regulated proteins and 262 down-regulated proteins) were significantly changed in HEV-infected HepG2 cells relative to in the Mock group. Significant changes in DEPs were found in HEV-infected HepG2 cells supplemented with PS (201 up-regulated proteins and 63 down-regulated proteins, Fig. 3A). Clustering analysis was performed to assess the expression profiles of 548 DEPs in HepG2 cells with or without PS treatment. Notably, HEV-infected HepG2 cells supplemented with PS showed significant up-regulation compared with mock or HEV-infected cells supplemented with FBS (Fig. 3B). Meanwhile, the PPI networks of DEPs were constructed to analyze the interactions of these proteins. The PPIs in the HEV versus Mock, HEV + PS versus Mock, and HEV + PS versus HEV groups consisted of 285 proteins and 2997 interactions (Fig. 3C), 240 proteins and 2227 interactions (Fig. 3D), 375 proteins and 4491 interactions (Fig. 3E), respectively. Up-regulated or down-regulated proteins tended to cluster together because of their PPIs.

**Fig. 4:**
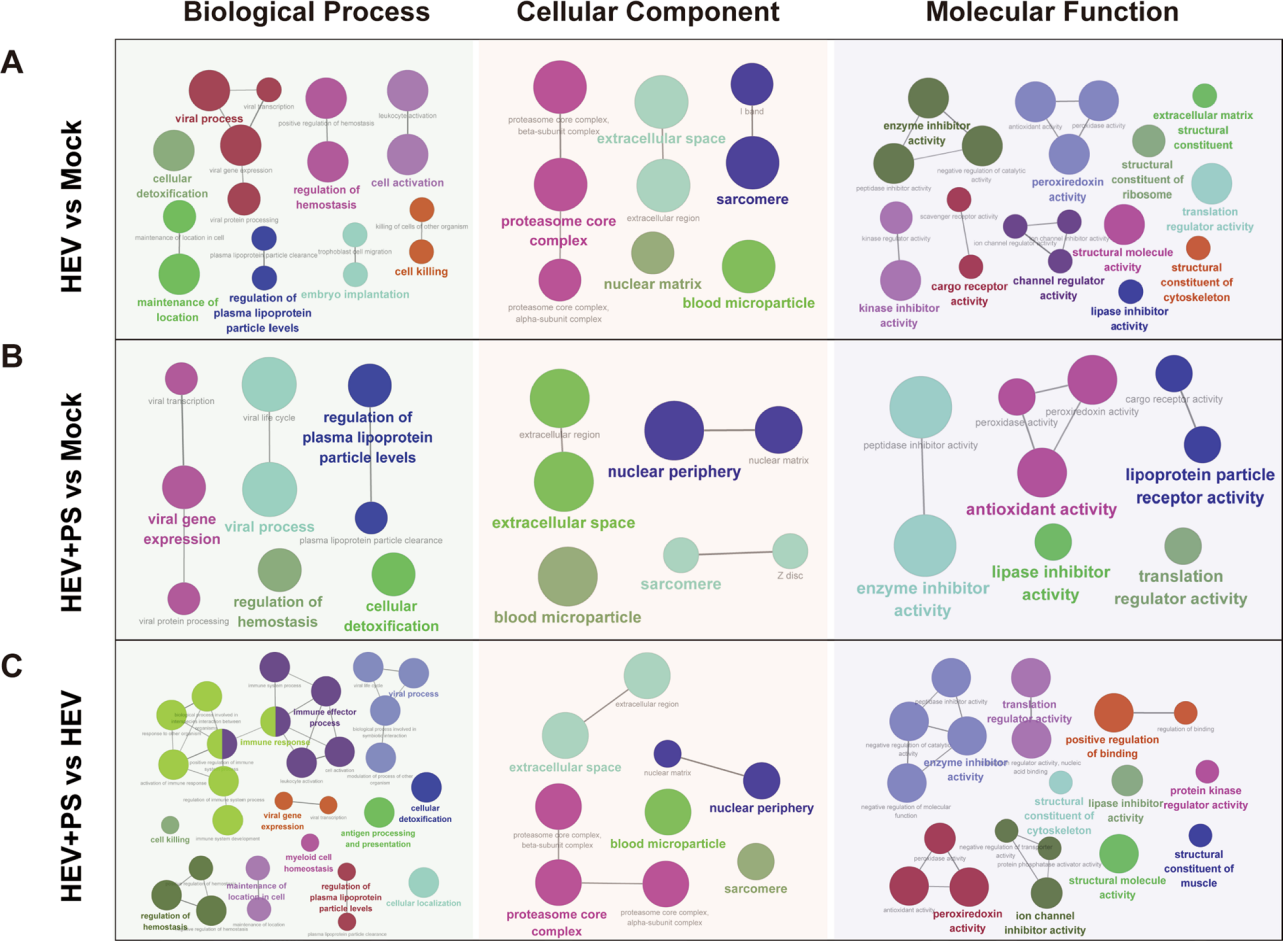
GO enrichment analysis based on Cytoscape software with ClueGO was used to reveal the differences and connections of biological process, cellular component, and molecular function terms enriched in the HEV versus Mock group (**A**), HEV + PS versus Mock group (**B**), and HEV + PS versus HEV group (**C**)

GO and KEGG annotation and enrichment analysis were performed on the DEPs to explore the functional changes between different groups. Remarkably, cargo receptor activity, channel inhibitor activity, peroxiredoxin activity, enzyme inhibitor activity, and translation regulator activity were significant changed in HEV-infected groups (supplemented with FBS or PS) compared with mock uninfected cells (HEV vs. Mock, Fig. 4A; HEV + PS vs. Mock, Fig. 4B), which indicated that HEV infection activate host defense reactions. Notably, a large number of DEPs involved in host immune responses, including activation of immune, regulation of immune system process, immune system development and leukocyte activation, were activated in HEV-infected cells supplemented with PS compared with those supplemented with FBS (HEV + PS vs. HEV, Fig. 4C), which implied that the changes of host immune responses during pregnancy may facilitate HEV replication.

**Fig. 5:**
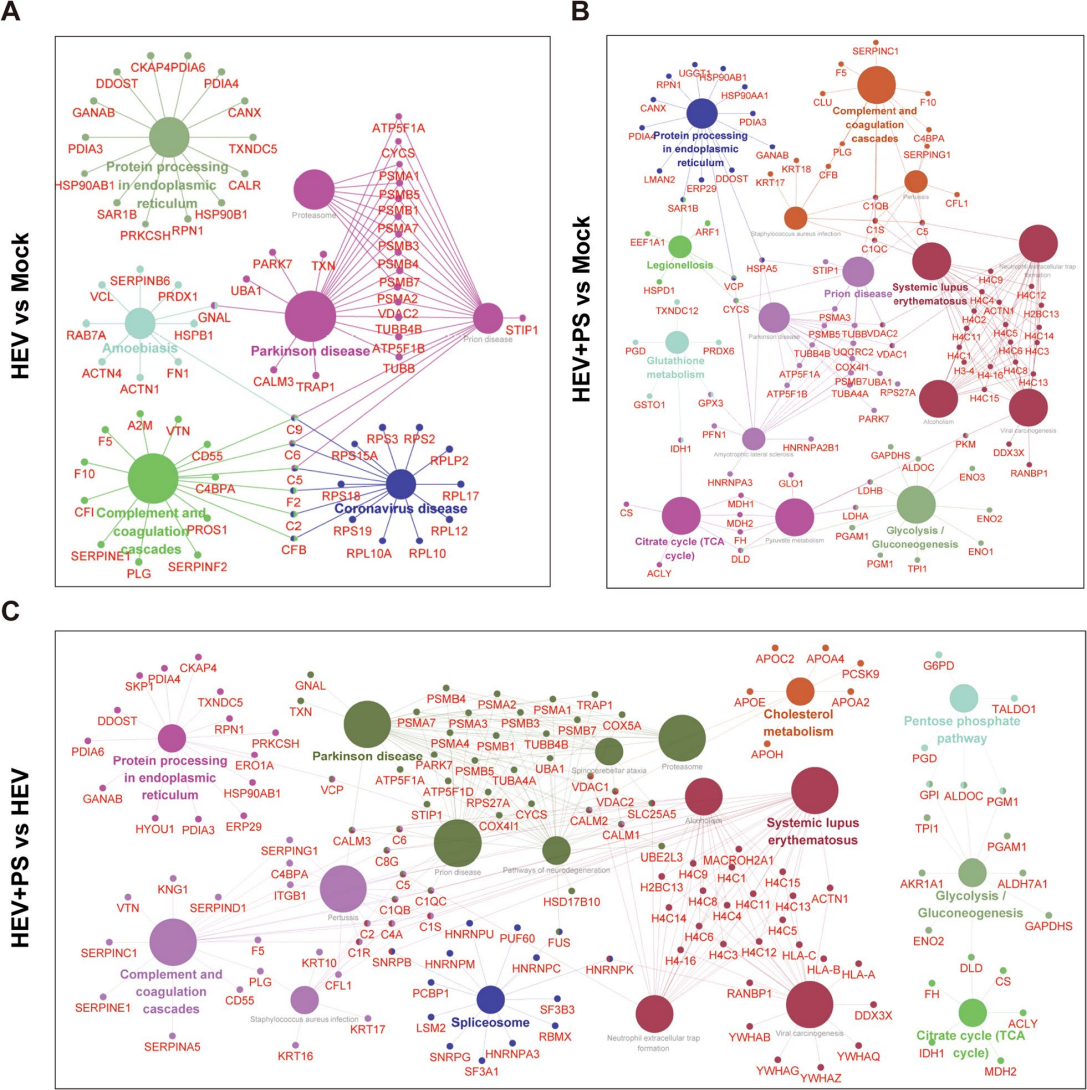
KEGG pathway analysis based on Cytoscape software with ClueGO was used to reveal the differences and connections of the significant signaling pathways enriched in the HEV versus Mock group (**A**), HEV + PS versus Mock group (**B**), and HEV + PS versus HEV group (**C**)

KEGG pathway analysis suggested that the signaling networks in HEV-infected cells supplemented with PS had increased compared with those supplemented with FBS. Compared with those in the uninfected Mock cells, these DEPs in HEV-infected cells supplemented with FBS were mainly involved in diseases, such as Parkinson’s disease, coronavirus disease, and amoebiasis, as well as in complement and coagulation cascades (HEV vs. Mock, Fig. 5A). These proteins in HEV-infected cells supplemented with PS also participated in some metabolic networks, such as the citrate cycle, glutathione metabolism, and glycolysis (HEV + PS vs. Mock, Fig. 5B). Once the HEV-infected cells were supplemented with PS, additional pathways, including human diseases, metabolic networks, and immune responses, became involved (HEV + PS vs. HEV, Fig. 5C). These results suggested that supplementation with PS accelerated virus–host interactions and regulated additional signaling pathways.

### DEPs validation

**Fig. 6:**
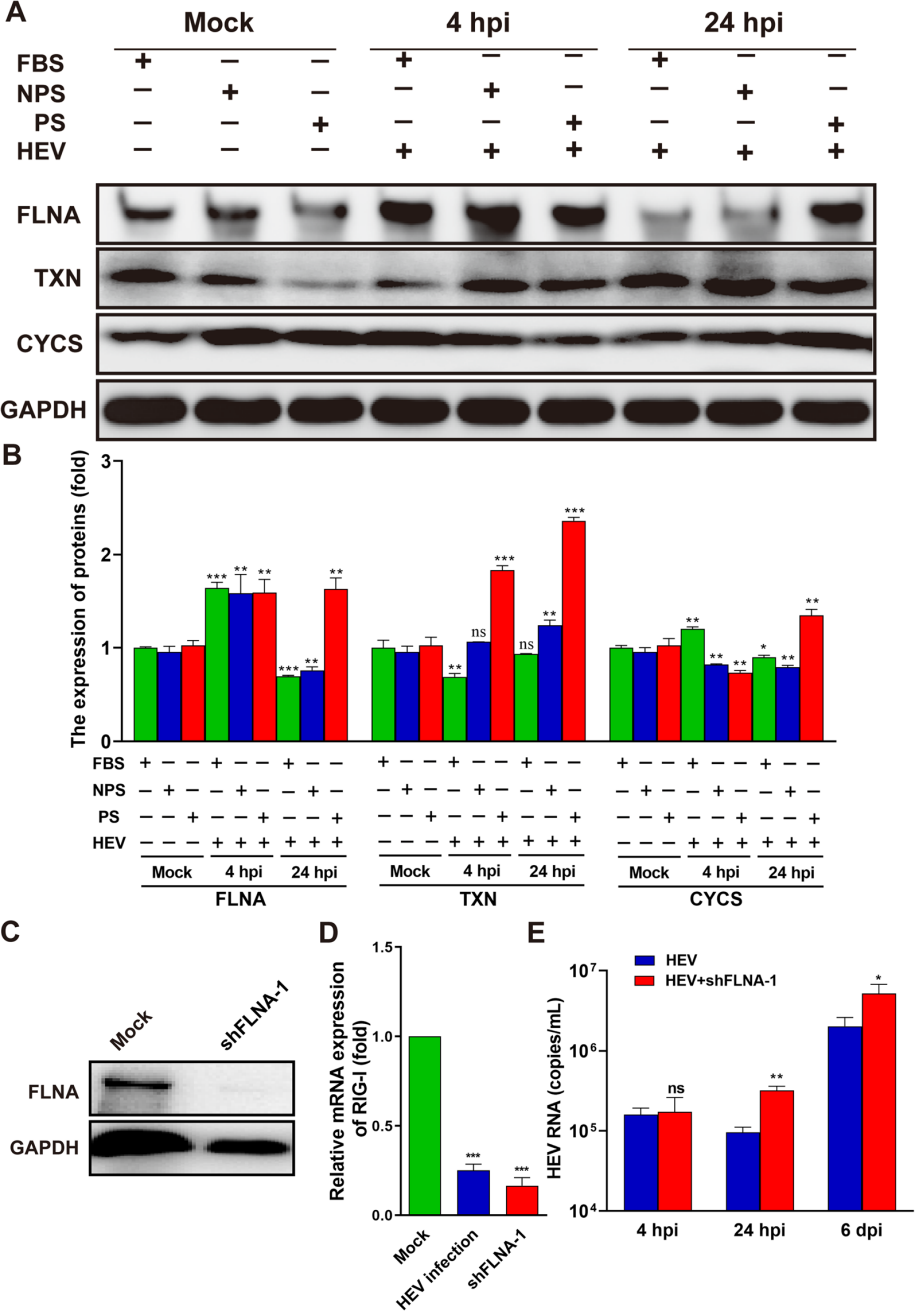
Validation of DEPs by Western blot analysis and qRT-PCR. **A** The expression levels of FLNA, TXN, and CYCS in Mock or HEV-infected HepG2 cells supplemented with FBS, NPS, or PS were analyzed through Western blot analysis at 4 and 24 hpi. GAPDH served as the loading control. **B** The relative expression levels of FLNA, TXN, and CYCS were analyzed and normalized to the expression level of GAPDH. **C** Knockdown of FLNA by shRNA validated by Western blot. **D** The relative gene expression of RIG-I in cells infected with HEV or transfected with shRNA targeting FLNA. **E** The copy number of HEV in HEV-infected cells transfected with or without shRNA targeting FLNA. Three independent experiments were preformed. Student’s *t*-test (two-tailed) was used to compare differences between two groups. *p < 0.05, **p < 0.01, ***p < 0.001

Three key proteins, including FLNA, TXN, and CYCS, were subjected to Western blot analysis at 0, 4, and 24 h post infection (hpi) to validate the iTRAQ process. The expression levels of the three proteins quantified by iTRAQ in cells infected with or without HEV are listed in Table 1. NPS isolated from healthy uninfected women was used as the control to further identify the effects of PS on HEV-infected cells. Notably, FLNA, an actin-binding protein that plays a primary role in signal transduction by linking the actin cytoskeleton to various transmembrane proteins to facilitate intracellular communication [33], was activated once HEV bound to the receptors on the host membrane at 4 hpi but was inhibited at 24 hpi after the complete entry of HEV (Fig. 6A and 6B), except cells supplemented with PS. FLNA has been reported to regulate the actin cytoskeleton to facilitate HCV [34] and HIV-1 [35] infection. To clarify the interactions between HEV replication and FLNA expression, FLNA were knockdown by shRNA (Fig. 6C). Notably, the down regulation of FLNA significantly suppressed the expression of retinoic acid-inducible gene I (RIG-I)-like receptors, the most important pathogen recognition receptors (PRR) during viruses infection (Fig. 6D). As a consequence, the suppression of RIG-I by FLNA inhibition facilitate HEV replication (Fig. 6E). Thus, PS supplementation maintained the expression of FLNA, which may be responsible for the promotion of viral replication (Fig. 6A, B and C).

TXN is a key component in the link between redox regulation and disease pathogenesis [36]. It mainly participates in the regulation and activation of NF-κB and other transcription factors and also displays antiviral, anti-inflammatory, antiapoptotic, and cell proliferative activities [37]. Nakamura [38] found that TXN plays a protective role against the influenza virus. The protective mechanism of TXN can be attributed to its potent antioxidative and anti-inflammatory activities. In the present study, TXN significantly increased in the HEV-infected HepG2 cells supplemented with PS at 4 and 24 hpi (Fig. 6A and B, Additional file 1). The increase in TXN expression in the HEV-infected cells revealed that TXN may be involved in early antiviral or anti-inflammatory responses. However, this involvement needs to be further identified.

CYCS regulates the intrinsic apoptotic pathway and is altered during viral infections. CYCS oxidase (COX) VIC is activated in the early stage of influenza virus infection [39]. Moreover, the X protein of HBV impairs the mitochondrial respiration chain and energy metabolism through interactions with COX subunit III [40]. HIV infection results in the dysregulation of CYCS [41]. The interaction of CYCS with the HEV capsid protein has been confirmed by using a yeast two-hybridization system [42]. In the present study, CYCS significantly decreased in HEV-infected HepG2 cells supplemented with NPS or PS, but increased in cells supplemented with FBS at 4 hpi, the early stage of infection (Fig. 6A and 6B, Additional file 1). Significantly increased CYCS were observed in HEV-infected HepG2 cells supplemented with PS, but significantly decreased in HEV-infected HepG2 cells supplemented with FBS or NPS at 24 hpi (Fig. 6A and B, Additional file 1). HEV ORF3 interacts with CYCS to protect against mitochondrial depolarization and death [43]. The promotion of CYCS in HEV-infected cells supplemented with PS may contribute to mitochondrial protection.

## Discussion

HEV infection is a serious global health issue. It causes high maternal case fatality rate (> 25%), miscarriages when infected with genotype 1 and 2 HEV during their late of pregnancy [44]. Although maternal death related to HEV infections were rarely reported in pregnant women who infected with genotype 3 or 4 HEV, adverse pregnancy outcomes such as abortion, preterm deliveries, stillbirths and perinatal case fatality rate were reported in China [8, 9]. However, the pathogenesis of HEV infection in pregnant women remains unknown.

We previously found that PS significantly facilitates HEV replication through the inhibition of estrogen signaling pathways in vitro [10]. Nevertheless, the virus–host interaction networks involved in this effect remain unclear. In general, viruses alter the signaling pathways of host cells to create a suitable environment for viral infection and replication. Therefore, comprehensive proteomic analysis should be performed to understand the host reactions against HEV infection. In the present study, HEV-infected cells supplemented with PS to simulate HEV infection in pregnant women in vitro to explore the mechanism of HEV infection during pregnancy. HEV-infected cells supplemented with NPS was omitted for proteomic analysis, because significantly elevated hormones are the most difference between pregnant women and non-pregnant women, and increased oestrogen and progesterone during pregnancy facilitates HEV replication has been identified in vivo and in vitro [12, 13]. Remarkably, HEV-infected cells supplemented with PS exhibited more up-regulated proteins and viral–host interactions than those supplemented with FBS. Especially, a large number of host immune–related proteins were activated in HEV-infected cells supplemented with PS analyzed by PPI, indicating that more viral–host interactions during pregnancy may facilitate HEV infection. GO functional analysis further identified that HEV infection during pregnancy is more active to regulate the host immune system than nonpregnat ones. The obviously increased viral–host interactions may be partly responsible for the severer symptoms in pregnant women than general population.

Up to now, the receptor of HEV is still unclear. The comprehensive proteomic analysis shown that the cargo receptor activity, channel inhibitor activity, structural constituent of cytoskeleton and the positive regulation of binding were activated in these HEV-infected cells supplemented with PS than those supplemented with FBS, which indicated that the supplemention of PS benefit the entry of HEV. FLNA is a cytoskeleton has been reported to regulate the actin to affect HIV and HCV replication [45, 46]. We found HEV invasion activated the expression of FLNA at early stage of infection, then inhibited its expression after entrance at 24 hpi. The supplement of PS activated the expression of FLNA, and the inhibition of FLNA facilitate HEV replication, which suggested that FLNA is closely associated with HEV infection, and further study should be performed in the future. Notably, the inhibition of FLNA suppressed the gene expression of RIG-I, a key antiviral ISG against HEV infection. Although significantly inhibited RIG-I and other ISGs had been observed in HEV-infected pregnant rhesus macaques [14], the detail interactions among HEV, FLNA and RIG-I is still unclear.

Pregnant women have to suppress their immunity to protect their fetuses from immunological recognition and rejection. Pregnant women with reduced immune response are susceptible to viral infection and adverse maternal–fetal outcomes [12, 13, 47, 48]. During pregnancy, the increase in hormones, especially progesterone and estrogen, activates numerous signaling pathways. The changes caused by the hormones may affect the life cycle of HEV, which should be further explored. In addition, the influences of cytokines and chemokines on HEV infection during pregnancy also should be concerned in the future.

## Conclusion

In conclusion, comprehensive proteomic analysis were performed in HEV-infected HepG2 cells supplemented with PS by iTRAQ to explore the pathogenesis of HEV during pregnancy. More virus–host interactions and immune-related proteins were observed in HEV-infected cells supplemented with PS than those supplemented with FBS, it may be partly responsible for the adverse pregnancy outcomes in pregnant women than in nonpregnant women. This study provide new and comprehensive insight for exploring the pathogenesis of HEV during pregnancy.

## Supplementary Information

**Additional file 1.** Supplementary Figure.

## Data Availability

The datasets generated during and/or analysed during the current study are available from the corresponding author on reasonable request.

## Abbreviations

HEV: Hepatitis E virus
PS: Pregnancy serum
NPS: Nonpregnant serum
FBS: Fetal bovine serum
iTRAQ: Isobaric tags for relative and absolute quantification
GO: Gene Ontology
KEGG: Kyoto Encyclopedia of Genes and Genomes
PPI: Protein–protein interaction
HAV: Hepatitis A virus
HBV: Hepatitis B virus
HCV: Hepatitis C virus
HIV: Human immunodeficiency virus
FC: Fold changes
IFNs: Interferons
ISGs: Interferon-stimulated genes
FLNA: Filamin-A
TXN: Thioredoxin
CYCS: Cytochrome c
qRT-PCR: Real-time quantitative reverse transcription PCR (qRT-PCR)
