# Supplementary material for: Quantification of host proteomic responses to genotype 4 hepatitis E virus replication facilitated by pregnancy serum
Source: Virol J. 2023 Jun 1;20:111. doi: 10.1186/s12985-023-02080-5 (PMC10233519; doi:10.1186/s12985-023-02080-5)
Supplement: Supplementary file 1 — Additional file 1. Supplementary Figure. [file 12985_2023_2080_MOESM1_ESM.docx]

Supplementary file to

Quantification of host proteomic responses to genotype 4 hepatitis E virus replication facilitated by pregnancy serum

Chao Cong ^1#^, Zhongyao Qian^1#^, Yi Li^1^, Yanhong Bi^1^, Qiuxia He^1^, Tengyuan Li^1^, Yueping Xia^1^, Liangheng Xu^1^, Houfack K Mickael^1^, Wenhai Yu^2^*, Jiankun Liu^3^*, Daqiao Wei^1^*, Fen Huang^1^*

^1^ Medical School, Kunming University of Science and Technology, Kunming, PR China

^2^ Institute of Medical Biology, Chinese Academy of Medical Sciences and Peking Union Medical College, Kunming, PR China

^3^ 920th Hospital of Joint Logistics Support Force of PLA, Kunming, PR China;

*Correspondence: huangfen6789@163.com

^#^These authors have contributed equally to this work^.^


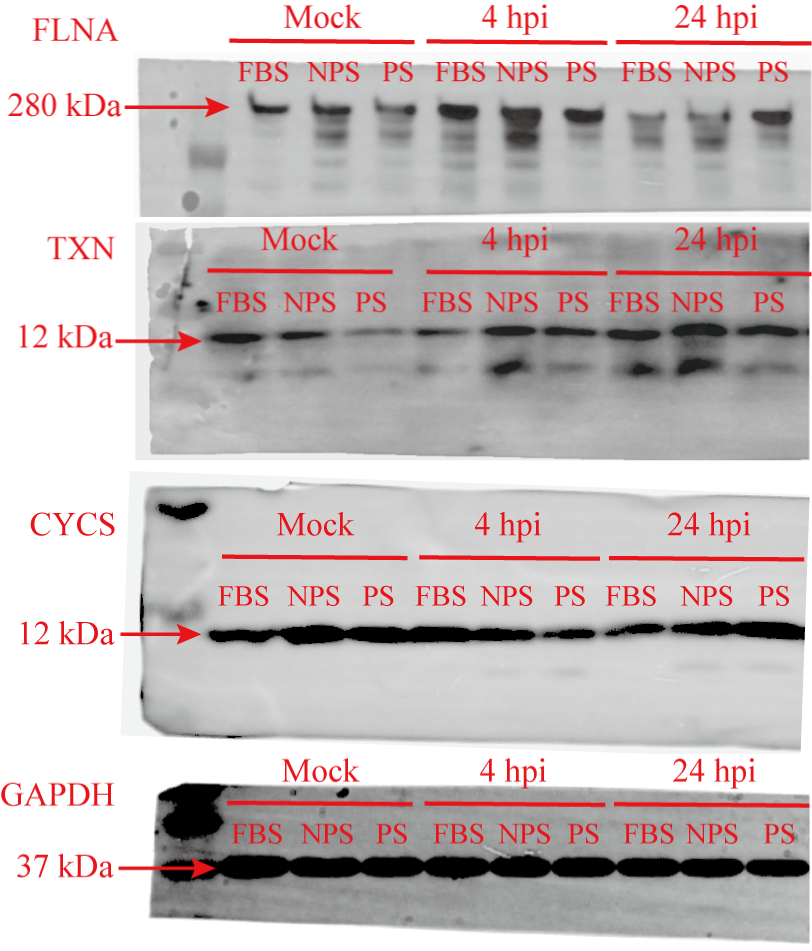


**Additional figure 1**. Western blotting analysis were performed to detect FLNA, TXN, CYCS and GAPDH in HEV-infected cells supplemented FBS, NPS or PS at 4 hpi and 24 hpi.
